# Simulation of intrafraction motion and overall geometric accuracy of a frameless intracranial radiosurgery process

**DOI:** 10.1120/jacmp.v9i4.2828

**Published:** 2008-10-24

**Authors:** Vladimir Feygelman, Luke Walker, Prakash Chinnaiyan, Kenneth Forster

**Affiliations:** ^1^ H. Lee Moffitt Cancer Center and Research Institute Division of Radiation Oncology Tampa Florida U.S.A.

**Keywords:** frameless stereotactic radiosurgery, intrafraction motion, clinical accuracy, optical tracking, PTV margin

## Abstract

We conducted a comprehensive evaluation of the clinical accuracy of an image‐guided frameless intracranial radiosurgery system. All links in the process chain were tested. Using healthy volunteers, we evaluated a novel method to prospectively quantify the range of target motion for optimal determination of the planning target volume (PTV) margin. The overall system isocentric accuracy was tested using a rigid anthropomorphic phantom containing a hidden target. Intrafraction motion was simulated in 5 healthy volunteers. Reinforced head‐and‐shoulders thermoplastic masks were used for immobilization. The subjects were placed in a treatment position for 15 minutes (the maximum expected time between repeated isocenter localizations) and the six‐degrees‐of‐freedom target displacements were recorded with high frequency by tracking infrared markers. The markers were placed on a customized piece of thermoplastic secured to the head independently of the immobilization mask. Additional data were collected with the subjects holding their breath, talking, and deliberately moving. As compared with fiducial matching, the automatic registration algorithm did not introduce clinically significant errors (<0.3 mm difference). The hidden target test confirmed overall system isocentric accuracy of ≤1 mm (total three‐dimensional displacement). The subjects exhibited various patterns and ranges of head motion during the mock treatment. The total displacement vector encompassing 95% of the positional points varied from 0.4 mm to 2.9 mm. Pre‐planning motion simulation with optical tracking was tested on volunteers and appears promising for determination of patient‐specific PTV margins. Further patient study is necessary and is planned. In the meantime, system accuracy is sufficient for confident clinical use with 3 mm PTV margins.

PACS number: 87.53.Ly

## I. INTRODUCTION

Frameless image‐guided stereotactic radiosurgery (SRS) has been widely investigated in recent years as an attractive alternative to the original frame‐based approach.^(^ ^1^ ^–^ ^7^ ^)^ Although SRS offers the possibility for fractionated treatments, is less invasive for patients, and is simpler logistically for the treatment team, the overall clinical accuracy of each system used for this radiosurgery method requires careful assessment. The two most important components of the assessment are the global hidden target test of inherent system accuracy (end‐to‐end test) and the evaluation of target motion.

A substantial amount of original research was devoted to the subject of clinical accuracy of SRS. However, the studies reported in the literature do not adequately answer all the questions pertaining to the practical clinical implementation of the BrainLAB (Feldkirchen, Germany) frameless SRS system (Novalis), particularly in terms of the overall accuracy of the whole treatment chain, as emphasized in Mack et al.^(8)^

The positioning accuracy studies for the Novalis unit either did not take delivery inaccuracies into account,^(9)^ ^,^ ^(10)^ did not use the frameless approach and true three‐dimensional (3D) evaluation of delivery inaccuracy,^(11)^ or were limited to a coplanar beam delivery arrangement.^(12)^ The X‐ray imaging–based studies of intrafraction movement of the patient's head inside the immobilization mask was reported for both the Novalis^(13)^ and CyberKnife^(14)^ ^,^ ^(15)^ (Accuray, Sunnyvale, CA, U.S.A.) systems. These reports are based on relatively infrequent X‐ray snapshots taken by the localization system. Such studies are typically performed during treatment. Although very useful for acquisition of population‐based statistics and for retrospective analysis, these studies show a great deal of variability between cases, and as a result, they have limited predictive value for individual patients, particularly for single‐fraction SRS. This limitation is particularly important for the Novalis system, in which imaging is less frequent than it is in CyberKnife, and to assure adequate target coverage in the presence of intrafraction motion, more reliance must therefore be placed on judiciously expanded planning target volume (PTV) margins than on frequent positional adjustments.

A number of investigators recognized the advantages of the real‐time tracking capabilities of optical systems for verifying patient positioning and at the same time acquiring ample statistical data on target movement.^(1)^ ^,^ ^(5)^ ^,^ ^(16)^ ^,^ ^(17)^ However, the implementation of optical tracking during treatment as described in those manuscripts requires the elimination of approximately one third of the immobilization mask to accommodate the bite block with infrared markers. Among the various examples of modern commercial immobilization systems described in the literature,^(^ ^18^ ^–^ ^21^ ^)^ not one appears to conform to such a configuration. Although real‐time position monitoring may be the ultimate tool for image‐guided therapy, and although the reported results are solid, some institutions are reluctant to make a substantial alteration that could potentially compromise the integrity of a commercially available immobilization mask. In particular, the immobilization set marketed by BrainLAB for frameless SRS extends to the shoulder level. Placement of the infrared markers for the typical commercially available mask is limited to the outside of the immobilization device, which underestimates the extent of target motion.^(13)^

We recently commissioned a frameless stereotactic image‐guided radiosurgery procedure on a 6‐MV linear accelerator equipped with a micro‐multileaf collimator and a Novalis Body/ExacTrac X‐ray 6D positioning system (BrainLAB). In the present paper, we evaluate the overall geometric accuracy of the whole frameless SRS process as implemented at our institution. The combined accuracy of the hardware and software of the positioning subsystem on similar units was characterized previously.^(9)^ That work showed that submillimeter accuracy for phantom positioning can be achieved. Repositioning accuracy^(19)^ and motion studies of mask exteriors^(13)^ and patients inside the masks^(^ ^13^ ^–^ ^15^ ^)^ for a variety of thermoplastic immobilization systems have been reported as well.

The geometric errors associated with targeting and delivery are addressed through verification of the performance of the subsystems and through the global hidden target system test. To evaluate the effect of head movement inside the commercially available immobilization device, we report the results of a volunteer study based on real‐time tracking of reflective markers secured on the patient's skull independently of the immobilization mask. This study was aimed at determining the feasibility of pretreatment simulation and quantification of expected movement for individual patients, without the need to substantially modify the standard immobilization device. Based on the estimate of all geometric uncertainties, we discuss a strategy for using patient‐specific PTV margins.

## II. METHODS

### A. Treatment system

Winston–Lutz tests^(22)^ recently performed on our system confirm that the radiation isocenter is confined to a sphere of 0.35 mm radius around the mechanical isocenter, with couch rotations included. The unit is equipped with a robotic ExacTrac 6D couch, allowing for translational and rotational setup corrections. The detailed commissioning procedure for the couch will be described separately. Image guidance is provided by the integrated positioning system known as Novalis Body/ExacTrac. A similar system was previously described in detail.^(6)^ ^,^ ^(9)^

### B. Open target test of the imaging chain isocentric accuracy

A plastic sphere of 5.9 mm diameter was glued to the end of a plastic rod. A 16‐slice Philips Brilliance scanner (Philips Medical Systems, Cleveland, OH) in helical mode was used to perform a computed tomography (CT) scan, with a reconstructed slice thickness of 0.8 mm and an increment of 0.1 mm. The reconstruction field of view was 180 mm, which corresponds to the pixel size of 0.35 mm. The spherical object was contoured as a target in the iPlan treatment planning software (ver. 3.0: BrainLAB), and the isocenter was placed in the center of that target. The sphere was carefully centered on the room lasers, which are known to intersect at the radiation isocenter of the accelerator. The digitally reconstructed radiograph (DRR) image filter settings of the software preclude good visualization of low atomic number objects. Instead, the overlaid target contours were used as reference images for registration. Radiographs were taken and manually registered to the plan data. The resulting table shifts were recorded and executed under infrared guidance.

Three separate CT scans of the small plastic sphere were performed. For each corresponding treatment plan, the object was aligned on the room lasers and imaged with kilovoltage X‐rays three separate times. Each image was analyzed three times by alternating observers, thus resulting in a total of 27 recorded shift vectors.

### C. Fiducial as compared with similarity‐measure image registration

We generated three different sets of DRRs of an anthropomorphic head phantom as described by Chuang et al.^(14)^ (ball‐cube phantom: Accuray). The phantom contains 4 fiducial markers visible on X‐rays. For each set of DRRs, image registration was performed a number of times. The manual fiducial‐matching algorithm and the automatic anatomic similarity‐measure algorithm were both carried out each time. Between measurements, the phantom was moved by 2 mm and 1 degree in various translational and rotational coordinates respectively.

### D. Global phantom hidden target test

**Figure 1:**
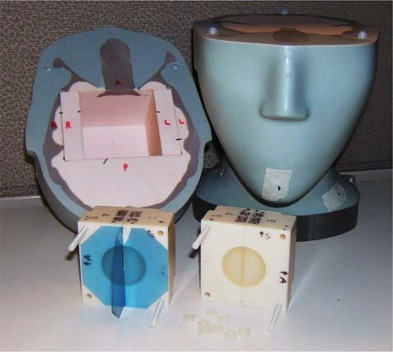
Disassembled ball‐cube phantom used for the global hidden target test.

A $63\;{\text{mm}}^{3}$ plastic cube was fitted tightly into the opening in the superior half of the ball‐cube phantom. A spherical target 31.5 mm in diameter was positioned in the middle of the cube. The cube was cut through the middle in two perpendicular planes to produce four quarters (Fig. 1). Two orthogonal square pieces of radiochromic film (Gafchromic EBT‐1417: International Specialty Products, Wayne, NJ) were placed in the cube, corresponding to the coronal and sagittal orientations. Two orthogonal edges of the film were carefully aligned to the edges of the cube, thus providing a known geometric relationship between the film and the target.

The phantom was scanned with the cranial radiosurgery protocol used at our institution: 1.5‐mm slice thickness, 250‐mm reconstruction field of view. The isocenter was placed in the center of the target, and a treatment plan was generated with 6 non‐coplanar arcs (BrainScan radiosurgery treatment planning software, ver. 5.31: BrainLAB). All beams were collimated with a tertiary 20‐mm SRS cone. The use of cones negates the need to analyze uncertainties in leaf positions. Beam weights were adjusted to produce the isocenter dose of 10 Gy, with a symmetrical dose distribution in all three principal planes.

The phantom was positioned on the treatment couch and a preliminary X‐ray alignment was done based on automatic bone anatomy registration to the DRRs. The phantom was adjusted to reduce angular misalignment to less than 0.3 degrees around all three axes. The alignment procedure was considered complete once residual translational errors were below 0.3 mm (approximately the size of 1 pixel of the X‐ray system detector), as suggested in Ryken et al.^(6)^

After irradiation, the radiochromic films were digitized in the transmission mode with a Microtek ScanMaker 9600XL flatbed charge‐coupled device digitizer (Microtek International, Husinchu, Taiwan). Most of the digitizer bed was covered with black paper to minimize the light scatter influence on the digitizer's relative sensitivity.^(23)^ Digitizer output was a 3×16‐bit red‐green‐blue tagged image format file at a resolution of 0.08mm/pixel. Only the red channel information was used. The images were analyzed with a spreadsheet routine based on ImageJ software (ver. 1.38x: public domain, available from http://rsb.info.nih.gov/ij/).

After customary background subtraction and filtering, a threshold pixel value was determined that would produce a region of interest (ROI) approximating a circle of 22 mm diameter positioned symmetrically inside of the irradiated area. The 22 mm diameter is the 70% isodose line on the treatment plan. The threshold function was applied to the image, and the resulting area was automatically contoured based on the difference between (binary) pixel values. The centroid of the contour is assumed to be the radiation isocenter. Finally, the distance from the centroid to the film edges and thus to the center of the target was determined. Because the films share the superior–inferior dimension, an average displacement from both was recorded. The anterior–posterior and lateral displacements were determined from the coronal and sagittal films respectively. The geometric accuracy of the edge extraction and distance measurements was validated by calculating the distance from an arbitrary point on the scanned image to the two opposite edges of the film, and by comparing the results to the physically measured film width. At less than 0.1 mm, this inaccuracy is negligible as compared with expected measured values.

The phantom was scanned on the CT scanner three times and, for each scan, the contouring, planning, and delivery procedures were repeated 3 times, generating a total of 9 film pairs that were analyzed.

### E. Immobilization

The immobilization system consists of a three‐piece extended thermoplastic clamshell mask (“frameless radiosurgery head and shoulders mask se”) registered to a carbon‐fiber base (BrainLAB). The three separate pieces of the mask are a perforated head support 3.5 mm thick, a solid anterior reinforcement layer with openings, and a second perforated anterior layer covering the head and shoulders. Additional thermoplastic material is used around the bridge of the nose.

### F. Intrafraction motion study

**Figure 2:**
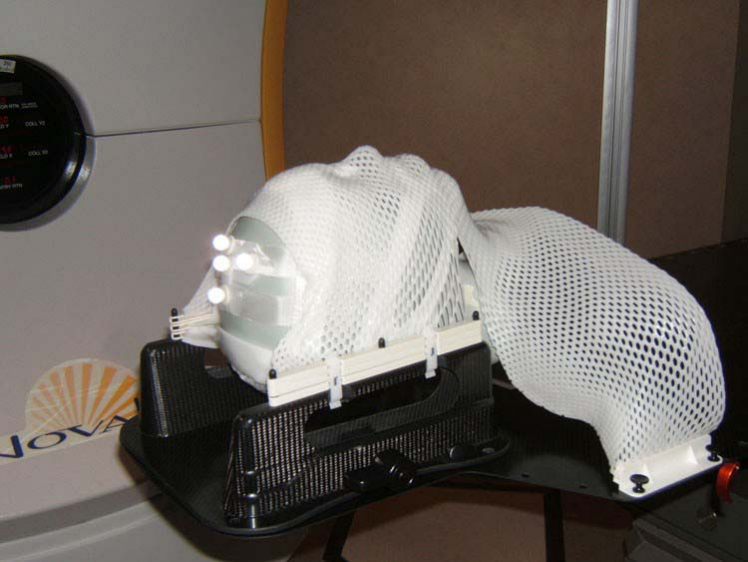
Immobilization device with infrared markers on the custom‐molded thermoplastic support, secured to the head independently of the mask.

The possible intrafraction head motion inside the mask was tested on 5 healthy volunteers of varying build and hair volume. Individual masks were constructed, and the volunteers were placed in the treatment position. An additional small thermoplastic piece was molded to the vertex of the head and secured using tape (Fig. 2). Four reflective markers were affixed to this additional piece. Because of the position of the markers, the table was rotated 90 degrees to assure robust image acquisition by the infrared cameras. The relative motion of the marker group was continuously monitored by the ExacTrac software and stored in a text file for offline analysis.

### G. General

Statistical analysis was performed using GraphPad Prism software (ver. 5.01: GraphPad Software, San Diego, CA).

## III. RESULTS

### A. Open target test of the imaging chain isocentric accuracy

Throughout the present paper, all shifts are reported as displacement of the *target* from the *radiation isocenter* of the accelerator. The positive directions of International Electrotechnical Commission coordinates *X, Y, Z* correspond, respectively, to the left, superior, and anterior anatomic directions if the patient is positioned supine with the head toward the gantry. Pitch, roll, and yaw correspond to rotations around the *X, Y*, and *Z* axes respectively.

For the open target test with a small object, Table 1 presents the mean displacement in all three axes, together with the 3D vector and descriptive statistics. These results are similar to 0.6±0.3mm, 0.5±0.2mm, and 0.7±0.2mm in the *X, Y*, and *Z* directions respectively (reported, for example, in Yan et al.^(9)^) or total displacements of 0.7 – 1 mm^(10)^ where different experimental setups were used. The distributions of displacement along all three axes were consistent with Gaussian (D'Agostino and Pearson normality test: p>0.35). Statistically, the mean deviation values for all three directions differ from 0 (one‐sample *t*‐test: p<0.0001), but the error in the *X* direction is effectively negligible (less than the acceptable residual alignment error of 0.3 mm and 0.3 degrees). The other two may be considered meaningful.

**Table 1:** Small plastic target isocentric positioning test^a^

*Displacement direction*	*Mean*	*Standard deviation*	*Min*	*Max*	*95% CI for mean*
LR (ΔX)	0.19	0.16	−0.05	0.50	0.06
SI (ΔY)	0.48	0.22	0.09	0.82	0.09
AP (ΔZ)	0.76	0.17	0.40	1.00	0.07
3D Vector	0.95	0.18	0.67	1.28	0.07

a N=27; all values in millimeters.

CI=confidence interval; LR=left–right; SI=superior–inferior; AP=anterior–posterior; 3D=three–dimensional.

### B. Fiducial as compared with similarity‐measure image registration

It was expected that an anatomic automatic image registration algorithm would perform well with the skull phantom because the similarity measure used for registration relies primarily on detecting sharp edges in the image,^(24)^ which are abundant in the bony anatomy of the head. The automatic registration algorithm has been tested against the fiducial registration procedure before,^(9)^ ^,^ ^(25)^ but we verified it for completeness. Table 2 presents the results of 27 measurements. Because the mean difference in each direction was less than the pixel size of the flat‐panel detector (0.27mm/pixel), we were satisfied that the anatomic automatic registration algorithm does not introduce any clinically significant systematic error as compared with the conceptually straightforward fiducial‐markers registration.

**Table 2:** Difference in isocentric positioning based on automatic bony anatomy registration as compared with manual matching of implanted fiducials^a^

*Displacement direction*	*Mean*	*Standard deviation*	*Min*	*Max*	*95% CI for mean*
LR (ΔX)	−0.18	0.12	−0.46	0.04	0.05
SI (ΔY)	−0.10	0.31	−0.42	0.62	0.12
AP (ΔZ)	0.13	0.13	0.20	0.39	0.05
3D vector	0.41	0.10	0.25	0.67	0.04

a N=27; all values in millimeters.

CI=confidence interval; LR=left—right; SI=superior—inferior AP=anterior—posterior; 3D=three—dimensional.

### C. Global phantom system test with a hidden target

The average ROI areas selected for radiation distribution centroid determination were $359.7\pm 19\;{\text{mm}}^{2}$ and $358.7\pm 24\;{\text{mm}}^{2}$ for coronal and sagittal films respectively. We verified that varying the ROI diameters between 21 mm and 23 mm (corresponding to a dose change of more than ±10% from the 70% level at 22 mm) did not change the centroid location by more than 0.1 mm. This result appears to differ from that of Vinci et al.,^(12)^ who found that the difference in isodose distribution displacement varied depending on whether the 70% or 80% dose level was chosen for analysis. The difference in average superior–inferior displacement extracted from the sagittal and coronal films was less than 0.1 mm, further indicating good internal consistency of the measurement routine.

Table 3 summarizes the target displacement results. The numbers reflect displacement of the target center from the center of experimental radiation distribution (radiation isocenter). Statistical distributions of values along all three axes are consistent with Gaussian (p>0.1). The deviations of mean *Y* and *Z* values from 0 are statistically significant (p=0.001 and 0.024 respectively) and can be considered meaningful. The error along the *X* axis is insignificant.

**Table 3:** Isocentric positioning errors from the global, anthropomorphic phantom system test^a^

*Displacement direction*	*Mean*	*Standard deviation*	*Min*	*Max*	*95% CI for mean*	
LR (ΔX)	−0.04	0.22	−0.44	0.31	−0.21	0.14
SI (ΔY)	−0.34	0.25	−0.68	0.03	−0.58	−0.20
AP (ΔZ)	−0.53	0.57	−1.37	0.34	−0.97	−0.09
3D vector	0.83	0.40	0.33	1.46	0.52	1.1

a N=9; all values in millimeters.

CI=confidence interval; LR=left—right SI=superior—inferior; AP=anterior—posterior; 3D=three—three‐dimensional.

### D. Intrafraction motion

**Figure 3:**
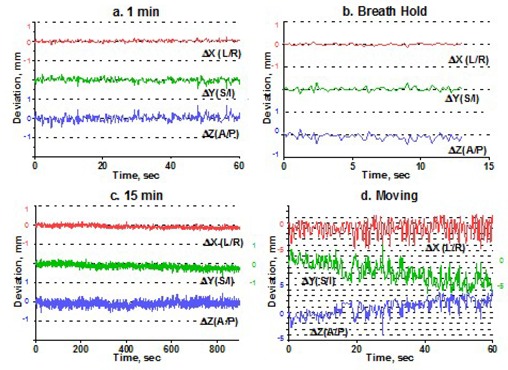
Subject 1, translational displacements of the head against time: (a) 1 minute of holding still; (b) breath hold; (c) 15 minutes of holding steady; (d) deliberately moving the head. For (a), (b), and (c), all displacements are from the average of the first 10 readings. Graph (d) shows raw data. Dotted grid lines correspond to 1‐mm intervals.

**Figure 4:**
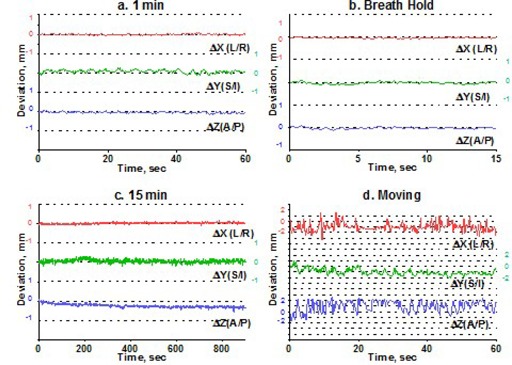
Subject 2, translational displacements of the head against time as described in Fig. 3.

**Figure 5:**
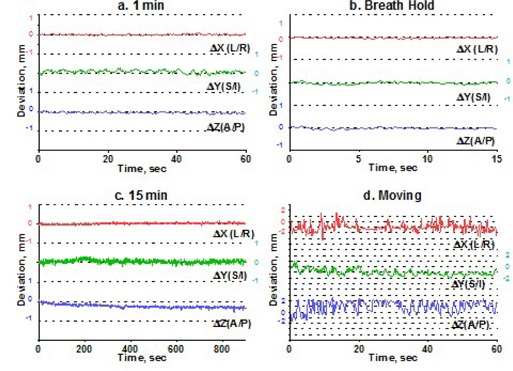
Subject 3, translational displacements of the head against time as described in Fig. 3.

**Figure 6:**
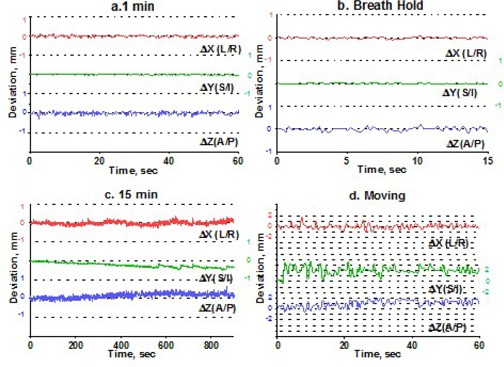
Subject 4, translational displacements of the head against time as described in Fig. 3.

**Figure 7:**
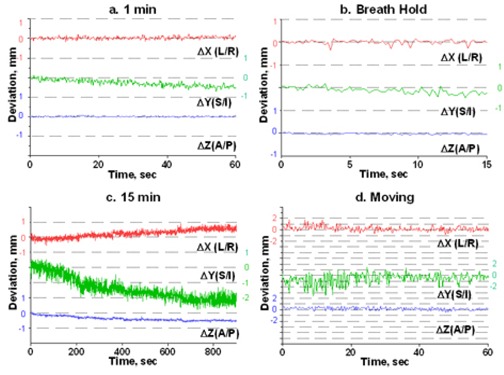
Subject 5, translational displacements of the head against time as described in Fig. 3.

**Figure 8:**
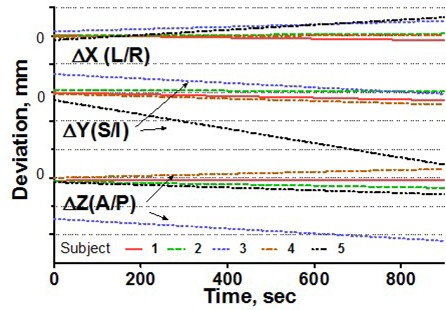
Linear regression of translational head displacements from the baseline for all subjects, 15‐minute treatment simulation. Dotted grid lines correspond to 1‐mm intervals.

To estimate intrafraction motion, we asked the volunteers to hold steady for 1 minute and then for 15 minutes. The 1‐minute time simulated the ability of a patient to hold still for imaging, and the 15‐minute time is a reasonable estimate of how long it takes to treat one isocenter with multiple arcs. In addition, a recent paper^(15)^ indicated that rather significant systematic shift in patient position may occur in a 15‐minute period. For multiple‐isocenter treatments, patients are re‐imaged between isocenter changes. We instructed the volunteers to deliberately move their heads inside the mask between the 1‐minute and 15‐minute measurements. This exercise served two purposes. First, it allowed us to estimate the maximum range of motion. Second, although repeating complete measurement sessions was logistically difficult, it helped to address the issue of immobilization consistency. There is no expectation of a significant patient shape change during the short period of time between the motion study and the radiosurgery treatment. If the patient were somehow in an unstable position inside the mask, the instability would likely have manifested itself in a significant difference between the 1‐minute study and the first minute of the 15‐minute study, given a substantial amount of deliberate intervening motion. Volunteers were also asked to hold their breath, so that we could assess baseline variability in the positioning of a marker group placed on a human subject. Except for the deliberate‐motion graphs, which plot the raw data, all displacements are reported from the initial position, defined as an average of the first 10 frames. Figs. 3 – 7 represent the translational displacements of the marker group attached to the head across time. Tables 4 and 5 summarize descriptive statistics for the motion studies (translational and rotational deviations respectively). The phantom data are included as a baseline. Linear regression lines for all 15‐minute runs are presented in Figs. 8 (translations) and 9 (rotations).

**Table 4:** Simulated intrafraction head movement in healthy volunteers (translational deviations)^a^ for breath hold (BH), 1 minute of holding still and 15 minutes of holding steady, talking, or deliberately moving the head

*Subj*.	*Experiment Cond*.	*Time (s)*	*N*	*Mean*	Δ*X(LR)*		*Max*	*Mean*	*Deviation statistics (mm) ΔY (SI)*			*Mean*	Δ*Z(AP)*		*Max*
					*SD*	*Min*			*SD*	*Min*	*Max*		*SD*	*Min*	
1	BH	13.2	66	−0.00	0.04	−0.10	0.11	0.03	0.09	−0.20	0.29	−0.08	0.14	−0.46	0.27
	1 min	60	301	0.02	0.01	0.02	0.21	−0.01	0.11	−0.48	0.28	0.02	0.17	−0.49	0.68
	15 min	900	1801	−0.08	0.08	−0.32	0.18	−0.14	0.13	−0.65	0.25	−0.08	0.15	−0.58	0.50
	Talking	N/a													
	Moving	60	301			−3.9	2.6			−3.8	5.2			−5.0	2.9
2	BH	16.4	82	−0.02	0.03	−0.09	0.04	−0.02	0.05	−0.14	0.08	−0.02	0.04	−0.13	0.07
	1 min	60.6	303	−0.01	0.03	−0.10	0.07	0.08	0.08	−0.15	0.27	−0.05	0.04	−0.16	0.08
	15 min	900	1801	0.03	0.04	−0.10	0.15	0.06	0.08	−0.19	0.33	−0.24	0.08	−0.60	0.07
	Talking	25	126	0.00	0.06	−0.14	0.13	0.00	0.09	−0.18	0.19	0.00	0.07	−0.19	0.16
	Moving	60	301			−2.3	2.6			−1.0	1.9			−2.7	1.6
3	BH	15.2	76	0.01	0.04	−0.08	0.10	0.00	0.03	−0.07	.08	−0.13	0.12	−0.41	0.09
	1 min	60	301	−0.01	0.04	−0.11	0.09	−0.04	0.04	−0.13	0.09	−0.43	0.26	−0.90	0.22
	15 min	900	1801	0.33	0.12	−0.12	0.66	0.30	0.40	−1.5	0.67	−1.8	0.49	−2.8	0.74
	Talking	19.8	99	0.02	0.12	−0.17	0.50	0.04	0.35	−0.41	1.0	−0.85	0.73	−3.1	0.24
	Moving	60	301			−1.6	1.5			−0.76	0.57			−2.0	4.2
4	BH	15.2	76	−0.01	0.05	−0.10	0.12	0.00	0.02	−0.03	0.05	0.00	0.09	−0.20	0.22
	1 min	60	301	−0.02	0.05	−0.15	0.12	0.00	0.02	−0.07	0.05	0.00	0.08	−0.29	0.18
	15 min	900	1801	−0.01	0.09	−0.28	0.36	−0.23	0.12	−0.50	0.07	0.16	0.13	−0.24	0.65
	Talking	60	301	−0.09	0.15	−0.44	0.42	−0.1	0.08	−0.29	0.16	0.08	0.13	−0.21	0.51
	Moving	60	301			−1.1	1.7			−2.4	1.6			−1.6	1.0
5	BH	15.2	76	0.00	0.09	−0.36	0.16	−0.12	0.13	−0.43	0.18	−0.02	0.03	−0.08	0.05
	1 min	60	301	0.03	0.07	−0.18	0.23	−0.28	0.17	−0.71	0.11	0.00	0.03	−0.07	0.08
	15 min	900	1801	0.25	0.26	−0.54	0.93	−1.4	0.75	−2.8	0.56	−0.37	0.14	−0.63	0.09
	Talking	60	301	−0.18	0.16	−0.82	0.17	−0.05	0.35	−0.99	0.85	0.13	0.15	−0.31	0.6
	Moving	60	301			−0.8	1.8			−3.6	1.4			−0.4	0.66
Phantom	1 min	60	301	0.00	0.03	−0.09	0.11	0.00	0.07	−0.15	0.24	0.00	0.06	−0.11	0.26

a For all volunteer experiments, except those with deliberate movement, deviations are calculated from the average of the first 10 frames. For deliberate movement, only the range is reported.

Subj.=subject; cond.=experimental condition; LR=left–right; SD=standard deviation; SI=superior‐inferior; AP=anterior‐posterior.

**Table 5:** Simulated intrafraction head movement in healthy volunteers (rotational deviations)^a^

*Subj*.	*Experiment Cond*.	*Time (s)*	*N*	*Mean*	Δ*Roll*		*Max*	*Mean*	*Deviation statistics (degrees) ΔPitch*			*Mean*	Δ*Yaw*		*Max*
					*SD*	*Min*			*SD*	*Min*	*Max*		*SD*	*Min*	
1	BH	13.2	66	−0.00	0.02	−0.04	0.04	−0.03	0.06	−0.20	0.13	0.00	0.01	−0.03	0.03
	1 min	60	301	−0.01	0.03	−0.16	0.06	0.02	0.08	−0.2	0.32	0.00	0.02	−0.09	0.07
	15 min Talking	900 N/a	1801	0.03	0.03	−0.07	0.13	0.02	0.07	−0.2	0.31	−0.01	0.02	−0.05	0.08
	Moving	60	301			−0.82	1.3			−2.9	1.9			−0.44	0.37
2	BH	16.4	82	0.01	0.03	−0.04	0.06	−0.02	0.05	−0.19	0.07	−0.01	0.02	−0.05	0.04
	1 min	60.6	303	−0.02	0.03	−0.11	0.05	0.06	0.07	−0.13	0.26	0.02	0.02	−0.05	0.08
	15 min	900	1801	0.05	0.03	−0.05	0.15	0.12	0.08	−0.15	0.53	−0.00	0.04	−0.09	0.10
	Talking	25	126	−0.00	0.04	−0.14	0.10	0.00	0.09	−0.20	0.22	0.00	0.03	−0.06	0.09
	Moving	60	301			−0.94	1.2			−1.9	1.6			−0.68	0.46
3	BH	15.2	76	−0.03	0.05	−0.14	0.05	0.12	0.12	−0.09	0.40	−0.01	0.03	−0.08	0.04
	1 min	60	301	−0.09	0.06	−0.19	0.03	0.27	0.18	−0.11	0.53	−0.05	0.03	−0.1	0.01
	15 min	900	1801	−0.83	0.18	−1.2	0.27	1.6	0.44	−0.5	2.5	−0.01	0.04	−0.12	0.15
	Talking	19.8	99	−0.22	0.25	−1.1	0.10	0.61	0.59	−0.23	2.5	−0.07	0.08	−0.35	0.05
	Moving	60	301			−0.66	1.1			−2.8	1.3			−0.28	0.26
4	BH	15.2	76	0.01	0.03	−0.06	0.08	−0.00	0.04	−0.09	0.08	0.00	0.03	−0.05	0.07
	1 min	60	301	0.00	0.03	−0.07	0.09	−0.00	0.04	−0.12	0.10	−0.00	0.03	−0.08	0.07
	15 min	900	1801	−0.02	0.04	−0.13	0.09	0.09	0.09	−0.18	0.36	−0.07	0.05	−0.21	0.14
	Talking	60	301	0.02	0.06	−0.12	0.20	0.05	0.11	−0.19	0.34	−0.03	0.08	−0.20	0.23
	Moving	60	301			−1.1	0.61			−1.3	1.6			−0.36	0.30
5	BH	15.2	76	−0.01	0.04	−0.10	0.15	0.06	0.08	−0.13	0.23	0.03	0.05	−0.07	0.19
	1 min	60	301	−0.01	0.04	−0.08	0.07	0.16	0.09	−0.02	0.33	0.05	0.04	−0.02	0.13
	15 min	900	1801	−0.09	0.12	−0.44	0.27	0.75	0.41	−0.34	1.5	0.35	0.21	−0.21	0.72
	Talking	60	301	0.08	0.08	−0.07	0.41	−0.00	0.18	−0.49	0.46	0.05	0.10	−0.23	0.32
	Moving	60	301			−0.88	0.21			−0.65	1.8			−0.17	0.62
Phantom	1 min	60	301	0.00	0.02	−0.06	0.05	−0.00	0.05	−0.11	0.19	0.00	0.01	−0.04	0.03

a Same experiments as in Table 4.

Subj.=subject; cond.=experimental condition; LR=left–right; SD=standard deviation; SI=superior‐inferior; AP=anterior‐posterior.

For the 15‐second breath‐hold experiment, representing the least movement expected from a volunteer, the standard deviations in all spatial dimensions are below 0.15 mm and are not meaningfully different from the phantom baseline, which represents the inherent precision of the infrared system (Table 4). The maximum range of motion along any of the three axes is also similar to that of the phantom experiment and is an order of magnitude smaller than the maximum possible range estimated in the deliberate movement experiment (0.7 mm as compared with up to 9 mm). The graphs for subject 1 exhibit more sporadic deviations, but we observed no meaningful differences between volunteer movements during the breath‐hold experiment.

Simulation of the clinical situation yields substantially different results. Based on Figs. 3 – 7, the subjects can clearly be divided into two groups. Subjects 1, 2, and 4 maintained a very steady head position during the 15‐minute recording period. The trend lines for all displacements do not deviate from baseline by more than 0.3 mm or 0.3 degrees. Similarly, no trends away from the baseline are seen on the 1‐minute translational graphs (Figs. 3, 4, 6). Consistent with the breath‐hold data, the increased range of motion during the 1‐minute run for subject 1 is observed particularly along the *Z* axis (total range of 1.2 mm for subject 1 vs. 0.2 mm and 0.5 mm for subjects 2 and 4). The ranges for subjects 2 and 4 are comparable to those of the phantom baseline. On the other hand, subjects 3 and 5 showed significant head movement during the 15‐minute experiment (Figs. 5 and 7). The maximum trend line value difference from 0 approached 2.2 mm along the *Z* axis for subject 3 and 2.5 mm along the *Y* axis for subject 5 (Fig. 8). The trends in the same direction can be seen for the corresponding graphs during the 1‐minute runs (Figs. 5 and 7). The abrupt shifts seen around the 800‐second mark for subject 3 may be attributed to this volunteer's propensity to fall asleep during the measurements.

The maximum range of motion does not predict trend during the 15‐minute experiment. For example, the largest overall range of deliberate motion was exhibited by subject 1 in the *Y* and *Z* directions: 9 mm and 7.9 mm respectively (Table 4). However, the corresponding lines in Fig. 8 do not show any meaningful trends away from the baseline, and the standard deviations for the 15‐minute runs are less than 0.2 mm. For subjects 1, 2, and 4, the translational range of deliberate motion substantially exceeds the corresponding limits for all other experiments (3 – 9 mm vs. <1 mm).

For subjects 3 and 5, whose position was less stable with time, the ranges for all experiments are comparable, and in case ofΔ*Y* for subject 3, the 15‐minute run exhibits an even larger range of motion than does deliberate movement (2.1 mm vs. 1.3 mm).

**Figure 9:**
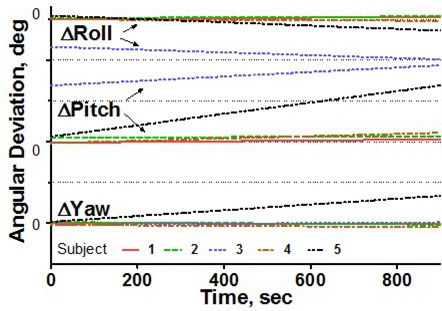
Linear regression of rotational head displacements from the baseline for all subjects, 15‐minute treatment simulation. Dotted grid lines correspond to 1‐degree intervals.

## IV. DISCUSSION

The open target positioning test confirmed that our system is capable of isocenter positioning accuracy of about 1 mm. This number compares favorably with previous reports^(9)^ ^,^ ^(13)^ and meets expectations once significant sources of error are taken into account. The two dominant sources of uncertainty are CT and X‐ray localization. For the protocol used, the overall 3D uncertainty with CT localization can be estimated at 0.5 mm. The Novalis X‐ray positioning system pixel resolution (0.27mm/pixel) leads to the estimated 3D localization uncertainty of 0.47 mm. Between the two, estimated precision is 0.7 mm, with contouring, X‐ray system alignment to isocenter, and the image registration procedure accounting for some additional error, bringing the overall uncertainty close to 1 mm.

The global hidden‐target phantom test confirmed that, when delivery errors are accounted for, the average 3D target displacement from radiation isocenter is still less than 1 mm. The difference between mean target displacement values for the small sphere (BB) and phantom tests is statistically significant (two‐tailed *t*‐test) for all axes (*X*: 0.2 mm, p=0.002 *Y*: 0.9 mm, p<0.0001; *Z*: 1.3 mm, p<0.001). The difference in the *X* direction is not considered meaningful. It does not appear that the BB test and the global phantom test indicate a systematic isocenter displacement in the same direction. We therefore used the results of the more comprehensive global hidden target test as the measure of the clinically relevant geometric accuracy of the system.

Although the inherent accuracy of the system is important and should be thoroughly evaluated, most publications, except for that by Chen et al.,^(3)^ point to target motion as the larger of the two major components of geometric uncertainty in frameless SRS. Studies of head motion in thermoplastic immobilization masks can be stratified by the frequency of positional verification and analyzed for their applicability to an a priori estimation of individual patient movement. Obtaining such an estimate before treatment planning would be beneficial for determining patient‐specific clinical target volume (CTV) to PTV expansion margins, as opposed to reverting to population‐based statistics.

Gilbeau et al.^(20)^ reported 2.2‐mm standard deviations of total displacement for a 4‐point fixation mask similar to the one used in our work. Their study used standard portal imaging applied weekly. Such a methodology is useful for acquiring population statistics, but cannot be used to prospectively estimate the range of motion for individual patients.

Linthout et al.^(13)^ used the six degrees of freedom registration of X‐rays to DRRs before and after treatment on a Novalis system as a measure of intrafraction movement. The authors acknowledged that with only two snapshots of patient position per fraction, it was not certain that the measured displacement was the largest that occurred during treatment. Fig. 5(c) can be used as a good hypothetical illustration of that statement. The snapshots at the beginning and the end of the simulated treatment would have resulted in a negligible reported deviation in the *Z* direction, but in reality, the target spent most of the 15 minutes more than 2 mm away from the original position. Also in Linthout et al.,^(13)^ the infrared markers were placed on the outer surface of the mask, and those authors concluded that range of motion based on marker tracking underestimates the actual range. Again, their methodology is more conducive to retrospective population‐based analysis than to prospective prediction of individual patient movement.

CyberKnife^(7)^ relies on more frequent position verification by orthogonal kilovoltage X‐rays. For intracranial^(15)^ ^,^ ^(26)^ and spinal^(14)^ ^,^ ^(15)^ ^,^ ^(27)^ radiosurgery, residual errors in patient position were sampled every 1 – 2 minutes. Although these studies provided more accurate time‐dependent information on patient motion, the data still have to be acquired during treatment. It was acknowledged^(14)^ that with X‐ray–based technology, it is difficult to predict the residual target errors for individual patients before actual treatment delivery. A suggestion was made^(14)^ ^,^ ^(26)^ to use more frequent imaging during the first fraction and then, depending on residual motion distribution, to adopt patient‐specific imaging frequency. Murphy et al.^(26)^ also noted that many patients exhibit systematic drifts in head position and that large (>2mm) position shifts are somewhat concentrated in a subset of the patient population that moves more often and by a greater amount than does the average patient. That finding further underscores the potential benefit of assigning patient‐specific, rather than population‐based, PTV margins—particularly for a system with relatively infrequent imaging such as Novalis. As a tool to study patient‐specific motion, X‐ray imaging is useful chiefly for managing treatment times or for customizing PTV margins for fractionated treatments on a specific type of radiosurgery system. In addition, it was reported^(26)^ that the patient motion and registration errors resulting from algorithm fluctuation around the optimal solution could not always be separated. Acquisition of robust motion statistics based on X‐ray imaging before treatment is not practical.

The University of Florida group was among the first to report the advantages of real‐time optical tracking in a clinical setup. The concept of attaching optical markers to a bite block was tested with radiosurgery patients. Originally,^(1)^ the frame was used to establish the reference position of the (light‐emitting) marker group. Later,^(28)^ the ExacTrac system with reflective markers was used for central nervous system and head‐and‐neck intensity‐modulated radiotherapy treatments. Treatment was interrupted and the patient manually repositioned when a displacement vector exceeded a predetermined threshold (2 mm). The maximum 3D displacement vector for all non‐patient subjects was reported as 1.5 mm for the 10‐ to 15‐minute runs. The volunteer population in the present work (Table 6) showed a greater range of motion (up to 2.9 mm total displacement vector).

**Table 6:** Total patient displacement (three‐dimensional vector) during 15‐minute run^a^

*Subj*.	*Mean*	*SD*	*Max*
1	0.3	0.1	0.8
2	0.3	0.1	0.6
3	1.9	0.5	2.9
4	0.3	0.1	0.8
5	1.5	0.7	2.9

a All values in millimeters.

Subj.=subject; SD=standard deviation.

Based on the data and methodology in Kim et al.,^(28)^ a standardized approach is justified, but the spread in our data led to a hypothesis that a predetermined population‐based PTV margin might be excessive for some patients and insufficient for others. This consideration is a particularly important one for intracranial radiosurgery, in which dose selection is strongly influenced by the target volume.^(29)^ On the other hand, real‐time optical tracking with the markers attached to a bite block is not compatible with the design concept of the BrainLAB radiosurgery head‐and‐shoulders immobilization system. This rather thick (3.5 mm) and further reinforced mask is designed to provide maximum rigidity and surface contact down to the shoulder level, with four fixation areas. To address this apparent challenge, we propose a simple compromise approach, which allows for individualized PTV margins. During mask fabrication, an additional cranial piece is made (Fig. 2). At least 24 hours after mask fabrication, a preplanning motion simulation study is performed. The cranial piece is secured to the patient's head independently of the mask. The patient is placed in the treatment position, and the mask is fastened in place. The table is rotated 90 degrees, four reflective markers are attached to the cranial piece, and a mock X‐ray alignment procedure is performed to establish the baseline position of the marker group. Then the patient is instructed to hold as steady as possible for about 15 minutes. The six‐degrees‐of‐freedom displacements are recorded every 0.5 seconds throughout the duration of the simulated treatment. Afterward, the maximum range of deliberate motion is also recorded. It is expected that, during actual treatment, no patient will spend more than 15 minutes without a repeat X‐ray registration procedure.

Initially, we assign a standard uniform PTV margin of 3 mm. Once our protocol is approved by the institutional review board, we plan to tailor margins to the individual patient. Numerous studies for various disease sites, a few of which are cited,^(^ ^30^ ^–^ ^38^ ^)^ analyze systematic and random target displacements during the course of treatment and attempt to provide margin recipes based on standard deviations. The use of population‐based statistics in various mathematical combinations is inherent in all these studies, which rely on relatively infrequent portal or CT imaging data with retrospective analysis. On the other hand, an optical system with its high acquisition frequency can generate statistically reliable data for every patient.^(16)^

Our approach to displacement analysis is similar to the uncertainty‐time histogram described in Kim et al.^(16)^ A histogram of this type is a plot of the accumulated time during which a patient stays within the corresponding movement uncertainty. We extract the data descriptors from the deviation plots of 15‐minute treatment simulation experiments. The underlying assumption is that the data acquired during the pretreatment simulation will closely represent future treatment. Although Chuang et al.^(14)^ cautioned against extrapolating the residual error data from one fraction, some support for this assumption can be found in Kim et al.^(16)^ The plots of cumulative time against overall displacement for 1, 10, and 20 fractions share similar sigmoid shapes, although the multi‐fraction curves are smoother, as expected. Also, the size of the displacement vector envelope encompassing 95% of the sampled points differed by no more than 0.2 mm between 5 and 20 fractions. The authors concluded that the 5‐fraction data could provide a meaningful gauge of patient movement uncertainty for the entire treatment duration. There is not much room in the SRS realm to acquire patient‐specific positional data from multiple fractions.

We selected the largest absolute value of the boundary of the range encompassing 95% of the data points ($\Delta_{95}^{d}$, where d=X Y, Z or3D) as the clinical measure of target displacement to be included in the margin calculation (Table 7). Substantial variability between the subjects is observed. For three volunteers, the value $\Delta_{95}^{3D}$ does not exceed 0.5 mm, which is close to the estimated image registration accuracy. For subjects 3 and 5 on the other hand, the displacements rise to the clinically significant levels of 2.6 – 2.9 mm. It is also instructive to compare the values of $\Delta d_{95}$ with twice the standard deviation for each respective dataset. For subjects 1, 2, and 4, who exhibited no significant trend away from the initial position, the difference between $\Delta d_{95}$ and 2o does not exceed 0.3 mm. However, for subjects 3 and 5, $\Delta d_{95}$ can substantially exceed the random variation.

**Table 7:** Hypothetical clinical target volume to planning target volume margins expansion (M) for the three axes components and in three dimensions (3D), based on root mean square of the system isocentric inaccuracy 8 and patient motion displacement $\Delta_{95}^{d}$ from the 15‐minute experiments^a^

*Subj*.	X *(LR)*		Y *(SI)*		Z *(AP)*		*3D Vector*	
	$\Delta_{95}^{x}$	$M_{x}$	$\Delta_{95}^{y}$	$M_{y}$	$\Delta_{95}^{z}$	$M_{z}$	$\Delta_{95}^{3D}$	$M_{3D}$
1	0.2	0.3	0.4	0.7	0.4	1.1	0.4	1.2
2	0.1	0.2	0.2	0.6	0.4	1.1	0.4	1.2
3	0.6	0.6	0.8	1.0	2.6	2.8	2.6	2.8
4	0.2	0.3	0.5	0.8	0.4	1.1	0.5	1.2
5	0.3	0.8	1.4	2.6	0.7	1.2	2.9	3.3

a All values in millimeters.

Subj.=subject; LR=left—right SI=superior—inferior; AP=anterior‐posterior.

Finally, to arrive at the CTV‐to‐PTV expansion margin, the inherent geometric uncertainty of the system and the error resulting from patient motion need to be combined in some fashion. We chose $\Delta d_{95}$ as the measure of uncertainty attributable to patient movement. Statistics for the global hidden target test are used to approximate the uncertainty resulting from the inherent geometric accuracy of the system (δ). To apply the (conservative) logic consistent with that used to quantify patient motion, we defined 8 as the value farthest away from 0 while still in the 95% confidence interval for isocenter displacement (Table 3). The resulting values are 0.2 mm, 0.6 mm, and 1 mm for *X Y*, and *Z* components considered separately, or 1.1 mm if the total displacement vector is analyzed. Because patient displacement is independent of inherent isocentric accuracy, those factors are added in quadrature to arrive at the hypothetical expansion margin *M* (Table 7). Note that the 3D margin is calculated from its own dataset and does not necessarily equal the root mean square of the components in the table.

The margins suggested so far (Table 7) do not take into account any errors associated with magnetic resonance (MR) image registration. One millimeter is a conservative estimate of the MR‐induced uncertainty reported in the SRS literature.^(8)^ ^,^ ^(39)^ ^,^ ^(40)^ Added in quadrature, it leads to suggested uniform PTV margins of 1.9 mm for subjects 1, 2, and 4; 3.2 mm for subject 3; and 3.3 mm for subject 5. This analysis is necessarily simplistic, because no treatment planning data exist. However, enough information potentially exists in a mock treatment study to construct non‐uniform individualized PTV margins when clinically warranted. Besides expected geometric accuracy, the margins can be influenced by achievable conformity of the dose distribution, target shape, and proximity of the critical structures.

## V. CONCLUSIONS

During commissioning of the Novalis frameless radiosurgery system, we thoroughly evaluated all potential sources of geometric error. The end‐to‐end hidden target test verified that, from CT scan to beam delivery, the system has submillimeter inherent accuracy.

A novel method is proposed to estimate the uncertainty associated with inevitable patient movement inside the immobilization mask. This method is based on a preplanning tracking study of infrared markers secured to the patient's head independently of the mask. A 15‐minute simulation run allows for a prospective estimation of the dimensions of the envelope that encompasses the target position for 95% of the treatment time. The dimensions of this envelope are used as a quantifier of target movement. Depending on clinical goals, enough information is available to construct either uniform or axis‐specific PTV margins for subsequent treatment planning.

The overall range of deliberate motion inside the mask does not necessarily predict the target movement during the 15‐minute run. The overall uniform margin that would have been proposed to a clinician is based on adding in quadrature the inherent system inaccuracy, target displacement range, and typical MR registration uncertainty. The resulting values range from 1.9 mm to 3.3 mm. Those results give us confidence that the overall process accuracy of frameless radiosurgery warrants initial clinical implementation with 3 mm uniform PTV margins. The initial data suggest that, in a subset of patients, combined 3D targeting uncertainty, excluding MR imaging, does not exceed 1.2 mm 95% of the time. Depending on the results of further study of motion patterns in an adequate number of patients, individualized treatment margins based on preplanning motion studies may be used in the future.

## ACKNOWLEDGMENT

The authors are indebted to the volunteers who endured the mask fabrication and treatment simulation processes.
